# Effectively engaging stakeholders and the public in developing violence prevention messages

**DOI:** 10.1186/s12905-017-0390-2

**Published:** 2017-05-11

**Authors:** Jennifer A. Boyko, C. Nadine Wathen, Anita Kothari

**Affiliations:** 1 School of Health Studies, Faculty of Health Sciences, London, Canada; 2 Faculty of Information & Media Studies, Western University, London, Canada; 3 Research Scholar, Centre for Research & Education on Violence Against Women & Children, Western University, London, Canada; 4 PreVAiL Research Network, http://www.PreVAiLResearch.ca

**Keywords:** Knowledge translation, Family violence, Research evidence, Public health messages

## Abstract

**Background:**

Preventing family violence requires that stakeholders and the broader public be involved in developing evidence-based violence prevention strategies. However, gaps exist in between what we know (knowledge), what we do (action), and the structures supporting practice (policy).

**Discussion:**

We discuss the broad challenge of mobilizing knowledge-for-action in family violence, with a primary focus on the issue of how stakeholders and the public can be effectively engaged when developing and communicating evidence-based violence prevention messages. We suggest that a comprehensive approach to stakeholder and public engagement in developing violence prevention messages includes: 1) clear and consistent messaging; 2) identifying and using, as appropriate, lessons from campaigns that show evidence of reducing specific types of violence; and 3) evidence-informed approaches for communicating to specific groups. Components of a comprehensive approach must take into account the available research evidence, implementation feasibility, and the context-specific nature of family violence.

**Summary:**

While strategies exist for engaging stakeholders and the public in messaging about family violence prevention, knowledge mobilization must be informed by evidence, dialogue with stakeholders, and proactive media strategies. This paper will be of interest to public health practitioners or others involved in planning and implementing violence prevention programs because it highlights what is known about the issue, potential solutions, and implementation considerations.

## Background

There is growing recognition among researchers, health and social system decision-makers, and the general public that as violence-related research continues to emerge it must be effectively mobilized to have desired impacts [1]. A starting point for learning about how to facilitate evidence uptake and use to orient health and social systems to prevent family violence includes examining specific knowledge mobilization strategies. The objective of this paper is to spark discussion about how to engage key stakeholders (i.e., researchers and decision-makers) and the broader public in developing and communicating, as one knowledge mobilization strategy, evidence-based violence prevention messages. We first discuss what is known about the problem of family violence as it relates to knowledge mobilization. Then we suggest potential components of a strategy aimed at engaging key stakeholders and the broader public in developing and communicating evidence-based violence prevention messages, as well as implementation considerations for such a strategy.

The knowledge mobilization strategy being discussed in this paper was identified and chosen by a steering committee of the Preventing Violence Across the Lifespan (PreVAiL) Research Network that was convened to help inform a deliberative dialogue (held as part of PreVAiL’s biennial team meeting) focused on how to mobilize research to orient health and social service systems to prevent family violence and its consequences [2]. PreVAiL is an international collaboration that brings together knowledge producers (i.e., researchers) and knowledge-user partners (i.e., policy actors from national and international organizations and governments with mandates that include violence prevention) to produce and share knowledge that will help children, women, and men exposed to violence. The steering group, which included partners from the Public Health Agency of Canada and the World Health Organization, met via teleconference to discuss challenges that could be explored through a deliberative dialogue process. The strategy discussed in this paper, along with three potential components of it, was chosen based on the informed opinion of the steering group and its potential to advance the field. The components were deemed potentially feasible and actionable for effectively engaging stakeholders in developing evidence-based family violence messaging. The steering group also believed the overall strategy and its components would effectively contribute to the broader challenge of mobilizing knowledge-for-action in family violence. An issue brief detailing relevant research evidence related to engaging stakeholders and the broader public in developing violence prevention messages was compiled as part of the deliberative dialogue process and is the basis for the evidence presented in this discussion paper.

## Discussion

### What do we know about the problem?

Three considerations frame the issue. The first is the understanding that violence is a multifaceted public health problem with multiple, often overlapping, causes and consequences (of victimization and perpetration), especially when taking a lifespan approach and understanding its intergenerational nature. Violence-related research often focuses on individual victims and perpetrators (though less so for the latter) rather than community and system-level or structural determinants and interventions. Inequities in violent acts that occur across social determinants of health (e.g., gender, age, ethnicity, culture) are magnified by disparities among high, middle and low-income communities and countries with respect to resources and capacity to address violence and its impacts [3, 4], which poses a significant challenge in terms of investing in any knowledge mobilization strategy.

The second consideration is the complex nature of violence as a problem remediable through public health interventions. Knowing “what works” (and doesn’t) and for whom (where, when) in existing programs, system arrangements and implementation strategies can be difficult. This complexity means that efforts to coordinate prevention require intersectoral, interjurisdictional and interprofessional collaboration. An example of such collaboration comes from Diadema, Brazil where municipal law and public health officials, alcohol establishments, and police worked together to successfully implement a law to prevent the selling of alcohol after 23:00, when complaints regarding violence against women are highest [5]. While there is emerging acknowledgement among international organizations (e.g., United Nations, WHO) that addressing system-level policy and structural factors that perpetuate violence is necessary [6, 7], collaborative efforts are rare and hampered by divergences in knowledge, practice, structures and resources.

The third consideration is that knowledge, practice and policy are shaped by the beliefs, values, norms and attitudes about violence entrenched in societies and cultures. As a result, some forms of violence may be condoned, justified or even glorified (e.g., hockey fights), and this impedes efforts to provide consistent messages about violent behaviour. In other words, many understandings and definitions of violence exist, adding to the lack of clarity regarding how best to tackle the problem. While there is often agreement about extreme acts (e.g., child sexual abuse), there are varying perspectives regarding acts perceived as less extreme (e.g., spanking), especially given different meanings and social constructions of violence held by individuals [8]. A comprehensive public health response to violence prevention would acknowledge and address deeply rooted precursors to violence.

These fundamental differences in how we define violence suggest that getting people to care about preventing it, and engage with messages, is a significant challenge. Violence-related messaging can result in seeing violence as other people’s problem and disengagement with the issue. While it may be helpful to combine or link different forms of violence for messaging (e.g., framing exposure to intimate partner violence as a form of child maltreatment), caution is also warranted so as not to minimize some forms of violence, or highlight some issues at the expense of others. The media can be a mechanism for research-based messages, and a tool to help generate public outcry that gets people to care. However, researchers do not necessarily have the skills they need for working with journalists, and vice versa, nor do media priorities necessarily match those of researchers or violence prevention advocates.

These three considerations demonstrate why effectively engaging stakeholders and the public in violence prevention knowledge mobilization is difficult. Simple messages are unlikely to be effective, and may offend or cause people to “tune-out.” There may also be strong backlash to certain messages. For example, men may react with injustice and anger in the form of anti-feminist campaigns if they feel unfairly blamed for the actions of others [9]. Similarly, parents with a positive attitude towards spanking may react with outrage to messages that equate all forms of corporal punishment with physical abuse [10]. While economic arguments such as the costs of violence to taxpayers, employers, etc. have become more common [11], these can overshadow the human toll of family violence. Three ways to address these issues are suggested next.

### Potential components of a strategy for communicating about family violence

The first proposed component is to develop consistent key messages regarding family violence. These might focus on the intergenerational nature of violence, the impacts of violence across the lifespan, including economic costs; and/or, the effects of children’s exposure to violence on their long-term development, physical and mental health, and socio-occupational functioning. We know from research that parents’ exposure to child maltreatment earlier in life is a key risk factor for their later perpetration of child physical abuse [12], and past experiences of family violence predict adult re-victimization [13, 14]. Furthermore, violence has negative physical and mental health consequences for victims across their lifespan [12]. Some evidence suggests that the most effective message strategy for male perpetrators of intimate partner violence is to focus on the impact of their behaviour on children [15], or on their employment [1]. Clear and consistent messages that reinforce these links may be very effective.

The second proposed component involves identifying and adopting lessons (with some caution) from similar campaigns, for example anti-bullying. A number of school-based anti-bullying programs have been shown to be effective [16–18], and some media campaigns focussed on bullying, cyber abuse and suicide have shown promise [17, 18]. However, while several anti-cyber abuse programs are promising, there is no definitive evidence that they reduce risky online behaviour (e.g., emailing strangers, revealing personal information) [19]. There is also no strong evidence regarding the effectiveness of media campaigns for bullying, cyber abuse, or suicide [17, 18]. In addition, the extent to which campaigns for other types of violence will be effective for family violence is unknown. That said, what is known about what should or should not be done in terms of violence prevention campaigns could be a useful starting place.

The third component is to identify, adapt or develop evidence-informed approaches for communicating to specific groups, i.e., health and social providers, policy actors, and the general public. Some campaigns focussing on intimate partner violence victims and perpetrators, for example, show promising results [20–22]; these could be modified for other groups, and evaluated. Approaches tailored to health and social service providers could build on a recent PreVAiL review of interventions to mobilize family violence evidence that found that a variety of strategies can be effective, at least in the short-term [1], but that the specific context of family violence must be considered [23]. Another review suggests internet-based interventions can be effective at promoting health behaviour change [24], an approach that might be useful for specific groups. It is important to note that while some approaches (e.g., media campaigns) for communicating to specific groups can change behaviour [25], the evidence regarding their effectiveness for child maltreatment and intimate partner violence prevention is inconclusive [26].

### Implementation considerations

The proposed components of a strategy for engaging stakeholders and the public in developing family violence prevention messages are more likely to achieve success if enablers and challenges to implementation are considered at the outset. In terms of enablers, lessons such as developing coalitions with diverse membership can be gleaned from other complex public health issues (e.g., smallpox eradication, tobacco control, HIV prevention) that have utilized social marketing campaigns. Just as important is an understanding of barriers to implementing evidence-based policy such as: poor communication between policymakers and researchers; insufficient evidence; mismatched timelines and goals; and conflicting or competing knowledge [27]. Media campaigns can also be costly to implement and evaluate, and using new technologies such as social media may bring in as yet unknown (positive and negative) consequences. Certainly an important challenge related to family violence knowledge is its emotional, and sometimes controversial, nature [22], and careful consideration must be given to the appropriateness of ‘remote’ modes of communication, including potential for harm and opportunities for support. In addition, communicating to policy actors requires an understanding of the policy process; interactive and iterative communication strategies [27, 28] are often required.

## Conclusion

Preventing family violence and its consequences is a complex public health challenge that requires the application of equally complex multi-component strategies. This paper discussed how to engage stakeholders and the public in developing violence prevention messages as one strategy for mobilizing knowledge-for-action to prevent family violence. The three potential components of such a strategy explored here are supported, to varying degrees, by research evidence and might be part of a comprehensive approach to mobilizing evidence about family violence, while at the same time continuing to address gaps in knowledge through research and evaluation. In moving any of the proposed components forward, public health actors should examine implementation feasibility from the outset, and ensure decisions related to knowledge mobilization are informed by research evidence, as well as context-specific considerations gained from a deep understanding of the nature of family violence and its impacts.

## Abbreviations

PreVAiL: Preventing Violence Across the Lifespan Research Network
